# Blood pressure risk factors in early adolescents: results from a Ugandan birth cohort

**DOI:** 10.1038/s41371-019-0178-y

**Published:** 2019-02-25

**Authors:** Swaib A. Lule, Benigna Namara, Helen Akurut, Lawrence Lubyayi, Margaret Nampijja, Florence Akello, Josephine Tumusiime, Judith C. Aujo, Gloria Oduru, Alexander J. Mentzer, Liam Smeeth, Alison M. Elliott, Emily L. Webb

**Affiliations:** 1 London School of Hygiene and Tropical Medicine, Keppel Street, London, WC1E 7HT UK; 2 MRC/UVRI & LSHTM Uganda Research Unit, P.O. Box 49 Entebbe, Uganda; 3 Department of Paediatrics, Mulago Hospital, P.O. Box 7051 Kampala, Uganda; 4 Wellcome Trust Centre for Human Genetics, University of Oxford, Oxford, UK

**Keywords:** Risk factors, Renovascular hypertension

## Abstract

We aimed to investigate life-course factors associated with blood pressure (BP) among Ugandan adolescents. Between 9th April 2003 and 24th November 2005, 2507 pregnant women from Entebbe municipality and Katabi sub-county were enrolled into a deworming trial. The resulting 2345 live-born offspring were followed to age 10 or 11 years, when between 20th May 2014 to 16th June 2016, BP was measured following standard protocols. Factors associated with BP were assessed using multivariable linear regression. BP was measured in 1119 adolescents with a median age of 10.2 years. Mean systolic BP and diastolic BP was 105.9 mmHg (standard deviation (SD) 8.2) and 65.2 mmHg (SD 7.3), respectively. Maternal gestational body mass index (BMI), higher maternal education status and family history of hypertension were positively associated with adolescent BP. Childhood (age ≤5 years) malaria was associated with lower adolescent systolic BP. Factors measured at time of BP measurement positively associated with systolic BP were age, BMI, waist circumference and *Trichuris trichiura* (whipworm) infection; higher vegetable consumption was associated with lower systolic BP. Results for diastolic BP were similar, except higher fruit, rather than higher vegetable consumption was associated with lower diastolic BP and there was no association with waist circumference or *Trichuris trichiura* infection. In summary, life-course exposures were associated with adolescent BP in this tropical birth cohort. Malaria early in life could impact later BP. Interventions initiated early in life targeting individuals with family history of hypertension, aiming to reduce adiposity (in pregnancy and adolescence) and promoting fruit and vegetable consumption might contribute to reducing the risk of high BP and subsequent cardiovascular diseases.

## Introduction

Once uncommon in Africa [1], high blood pressure (BP) and cardiovascular diseases (CVDs) have escalated on the continent over recent decades [2], affecting populations at younger ages than in more affluent countries [3], The rising burden of high BP in Africa has been attributed to a transition from active to more sedentary lifestyles and a rise in unhealthy dietary practices [2]. Data on individual level BP risk factors in African adolescents and children are sparse.

Although high BP is less common in children and adolescents than in adults, it initiates early in life, persists into adulthood [4] and predicts adulthood hypertension [5]. Diagnosis of CVDs is uncommon until middle-age, yet its antecedents, mainly cardiovascular and metabolic changes, begin early in life [6]. Globally, the high BP burden in younger age groups has risen [7], with estimated prevalence of 1–25% among African children and adolescents [8].

Severe persistent high BP is associated with increased risk of stroke and heart failure [9]; treatment reduces long-term sequelae [9]. In children and adolescents, high BP is often asymptomatic and unnoticed, despite international recommendations for regular BP measurement from three years of age [10]. Hypertension diagnosis is commonly missed or inaccurately classified in children and adolescents [11]. Consequently, over 75% of high BP among children and adolescents remains undiagnosed worldwide [12].

Earlier studies, mainly in adults, have demonstrated the role of established risk factors for high BP such as obesity [13] and physical activity [14]. There is little literature on childhood and adolescent BP determinants from Africa; in particular the impact of childhood infections (of special importance in Africa) remains understudied and unknown.

Childhood and adolescence are opportune periods for high BP control or prevention before clinical manifestation of hypertension or related CVDs. Identification of life-course BP risk factors unique to Africa is needed for the development of appropriate BP control strategies. We used longitudinally collected data from the Entebbe Mother and Baby Study (EMaBS), a large tropical birth cohort, to describe factors associated with adolescent BP.

## Methods

### Study design, setting and population

This longitudinal observational study investigated perinatal and life-course factors associated with BP among adolescents born in Wakiso district, Uganda. The EMaBS was a randomised double-blind placebo-controlled factorial trial [ISRCTN32849447], designed to investigate effects of worms and their treatment in pregnancy and childhood on response to childhood vaccines and on infections [15].

The study was conducted in Entebbe municipality and Katabi sub-county (a peninsula on the northern shores of Lake Victoria). In 2003–2005, 2507 women attending Entebbe Hospital antenatal clinic, in their second or third trimester were invited, enrolled and randomised to receive albendazole (400 mg) or placebo and praziquantel (40 mg/kg) or placebo [15].

Data were collected prenatally from women and resulting 2345 live-born offspring followed from birth. As previously described [16], at 15 months offspring were randomised to receive quarterly single-dose albendazole or placebo up to age five years. Disease events were recorded at the study clinic annually and when the child reported to the clinic with an illness. Children continued under follow-up (seen at routine annual visits and when sick) after trial completion. Between 20th May 2014 and 16th June 2016, additional data, including BP measurements, anthropometry, puberty, physical activity and diet were collected from 10- and 11-year-olds. Enrolment into the BP study was postponed for those with malaria (fever with malaria parasites) or other illness until they were well after being treated by the study team. Clinic based field workers conducted home visits and telephone calls to remind participants of their annual visit and also invite them to participate in the BP study. Participants who then attended their 10- or 11-year annual visit during the BP study period were then invited to enrol and take part in the BP study at that visit. Adolescents participated once, on their first 10 or 11-year annual visit occurring during the study period.

### Study procedures

Birth weight was measured and recorded immediately after birth in Entebbe hospital or obtained from child health cards for deliveries conducted elsewhere [17]. Weight and height at 10/11 years were measured with scales (Seca, Hamburg, Germany) and stadiometers (Seca 213, Hamburg Germany), respectively. Waist circumference was measured to the nearest 0.1 cm using a Seca tape measure (Seca 201, Hamburg, Germany). Body mass index (BMI) was calculated as weight in kilograms (kg) divided by height squared (m^2^). Trained clinicians examined and performed Tanner staging as described elsewhere [18].

Whole-genome genotyping of 1391 EMaBS samples was conducted at the Wellcome Trust Sanger Institute using Illumina HumanOmni2.5M-8 (‘octo’) Beadchip arrays, version 1.1 (Illumina Inc., San Diego, USA). Sickle-cell trait was imputed using a merged 1000 Genomes and African-specific reference panel [19].

For participants taking part in the BP study from the 21st January 2015 to 23rd December 2015, extra data on fat mass (FM), fat-free mass (FFM) and total body water mass (TBW) were collected by trained nurses using a segmental body composition analyser machine (SBCAM) [TANITA BC-418, TANITA Corporation, Tokyo Japan]. Briefly, participants stood barefooted on the posterior electrode base while holding two anterior electrodes handles of the SBCAM. Fat mass index = FMI (kg)/height (m^2^), fat-free mass index = FFMI (kg)/height (m^2^) and total body water mass index = TBWI (kg)/height(m^2^) were computed.

Stool and blood samples were collected from women at enrolment and annually from children. Stool was examined for helminth (*Schistosoma mansoni, Necator americanus, Ascaris lumbricoides and Trichuris trichiura*) ova and *Strongyloides* larvae using Kato-Katz [20] and charcoal culture [21] methods, respectively. Blood was examined for malaria parasites using Leishman’s stains [16]. Modified Knott’s method [22] was used for *Mansonella perstans*. Maternal HIV status at enrolment and children’s HIV status after 18 months of age were assessed using a rapid serial testing algorithm described elsewhere [21, 23]. In infancy, HIV status was determined using polymerase chain reaction [21].

At the 10- or 11-year annual visit, three BP measurements (at ~5 min intervals) were taken after 5 min rest using automated devices (Omron M6), with appropriate sized cuffs [5], by trained nurses following standard protocols described elsewhere [17].

For clinical care purposes, means of the three systolic BP and three diastolic BP measurements were calculated and BP percentiles determined using Centre for Disease Control height charts and 2004 updated National Health and Nutrition Examination Survey BP tables specific for sex, age and height [5, 10]. Those with mean systolic BP or diastolic BP ≥95th percentile (“high BP”) had their BP re-measured on up to two extra days, 1–2 weeks apart. “Pre-hypertension” was defined as systolic or diastolic BP ≥90th but <95th percentile. Those with persistent high BP on three different days were referred for specialist attention. Lifestyle modification was recommended for participants with systolic or diastolic BP ≥90th percentile.

For data analysis purposes, the means of the second and third systolic/diastolic BP readings on day 1 were used: day 1 second and third BP readings were lower than the first BP reading but similar to each other [17].

Ethical approval was granted by the Uganda Virus Research Institute Science and Ethics Committee; the Uganda National Council for Science and Technology; and the London School of Hygiene and Tropical Medicine. Written informed assent and consent were obtained for study participation.

### Statistical methods

Data were collected on pre-coded questionnaires and analysed with Stata 14.2 (College Station, TX, USA). Chi-squared tests (for categorical variables) and *t*-tests (for continuous variables) were used to compare characteristics of cohort members who participated and did not participate in the BP study.

Study outcomes were mean systolic BP and mean diastolic BP, based on the second and third day-one measurements. The decision was made to model these two continuous BP outcome variables rather than to dichotomise outcomes (for example, into normal versus hypertensive) as an analysis using these binary outcomes would be underpowered. Maternal, perinatal and offspring life-course factors considered as exposures and potential confounders were: maternal and adolescent socio-demographic and anthropometric characteristics; EMaBS trial interventions (praziquantel or albendazole); sickle-cell trait; illnesses and infections from birth to time of BP measurement; and body composition, puberty stage, diet, sleep pattern and physical activity at time of BP measurement. Area of residence was grouped into urban versus rural area using zones based on topography and settlements generated from geographical positioning system data [24]. Household socioeconomic index was generated using principal components analysis of building materials, household size and items owned [23]. Birth season was dichotomised into dry (rainfall below monthly median) and wet (rainfall above monthly median) season. Malaria infection in childhood (age ≤5 years) was investigated as clinical malaria (history of fever within the last 48 h or axillary temperature ≥37.5 °C and parasitaemia) and asymptomatic malaria (parasitaemia without fever at any annual visit up to 5 years). Information on diet was obtained as the number of days in a typical week over the previous month for which a given food was consumed. Puberty was grouped into pre-pubertal (stage 1) or pubertal (stages 2–5) for breast or pubic hair development using Tanner methods [18].

**Fig. 1:**
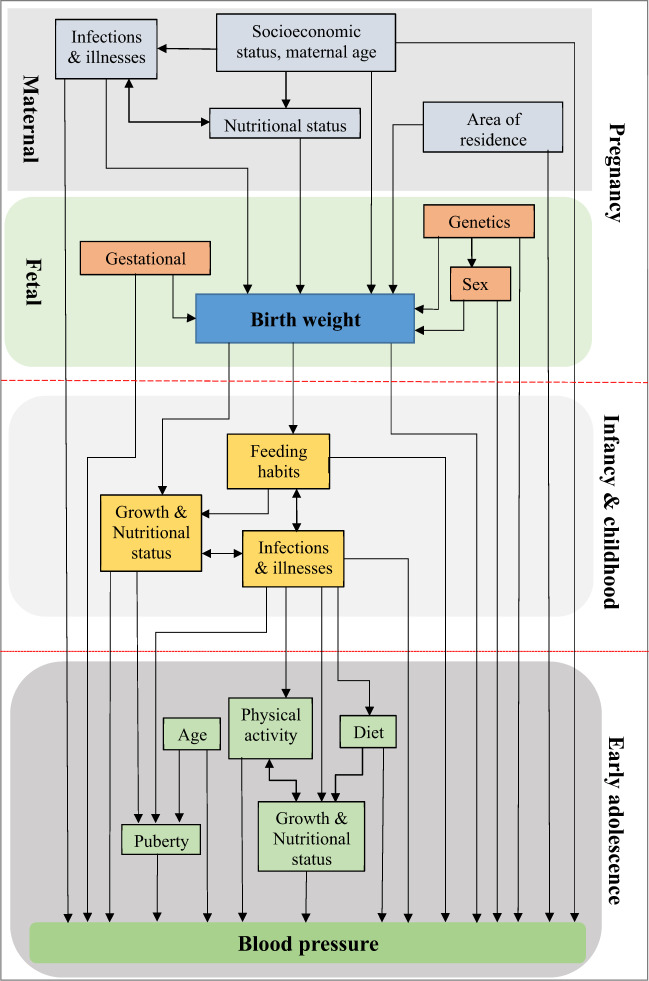
Conceptual framework

Linear regression analysis was used. Data satisfied the assumptions for linear regression. Crude associations were examined for each covariate and a 20% significance level was used for selecting covariates for multivariable models. Adolescents’ sex, age and BMI were confounders a priori. Multivariable analysis followed a hierarchical causal approach adding factors sequentially (Fig. 1).

Because of a large proportion of missing data, puberty and body composition variables were not included in model building for other exposures but their effects were each adjusted for variables included in the final multivariable model. Multicollinearity was assessed by considering the change in standard error, when potentially multicollinear variables were included in the same model.

## Results

### Participant characteristics

A total of 1119 EMaBS participants were enrolled into the BP study: 583 (52%) were males; 1100 (98.3%) singletons; 18 (2%) HIV positive; and 344 (31%) mixed feeding by 6 weeks. EMaBS adolescents participating in the BP study were similar to non-participants, except that mothers of participants were more likely to be of higher education status or married/cohabiting; offspring were less likely to be HIV positive or of a multiple birth, details published earlier [17].

At age 10/11 (median participant age 10.2 years (interquartile range (IQR): 10.0–10.9)), 117 (11%) were attending boarding schools, 441 (72%) were pre-pubertal stage for pubic hair development and 178 (65%) of girls were pre-pubertal stage for breast development. Mean BMI was 15.8 kg/m^2^ (standard deviation (SD) 1.9) and mean waist circumference 58.1 cm (SD 4.9). Body composition data were available for 176 (16%) participants, with mean fat mass index 2.9 kg/m^2^ (SD 1.2), fat-free mass index 12.8 kg/m^2^ (SD 1.4) and total body water mass index 9.5 kg/m^2^ (SD 0.9).

Over the previous month, starchy staple foods, animal proteins, fruit, vegetables and sugar drinks were consumed on average for 6.9 days/week (SD 0.8), 2.2 days/week (SD 1.7), 3 days/week (SD 2.2), 3.4 days/week (SD 2.3) and 1.7 days/week (SD 2.1), respectively. Nearly all adolescents (98%) reported adding salt to cooked food.

Mean systolic BP was 105.9 mmHg (SD 8.2) and mean diastolic BP was 65.2 mmHg (SD 7.3). There was no difference in mean systolic BP (*P*-value = 0.971) or diastolic BP (*P*-value = 0.141) between males and females. None of the adolescents had had a prior BP measurement or high BP diagnosis.

### Prevalence of high blood pressure

Using day 1 BP readings, the prevalence of pre-hypertension and high BP was 63 (10.8%) and 42 (7.2%), respectively, among males, and 54 (10.1%) and 52 (9.7%), respectively, among females. After extra measurements on the second and third visits and taking loss to follow-up into account, pre-hypertension prevalence was estimated as 2.2% in males and 0.7% in females; high BP prevalence was 0.4% in males and 1.8% in females.

### Risk factors for high blood pressure

Tables 1 and 2 show the relationship between examined characteristics and BP (systolic or diastolic) in adolescents. Maternal factors crudely positively associated with adolescent systolic BP were gestational BMI and education status; both remained associated with systolic BP after adjustment for other maternal factors. The trial interventions during pregnancy (albendazole and praziquantel) and early childhood (albendazole) had no effect on systolic or diastolic BP.

**Table 1:** Factors investigated for association with systolic blood pressure among adolescents from the Entebbe Mother and Baby Study (*N* = 1119)

Factors		Mean BP (SD)	Crude *β* (95% CI)	*P*-value	Adjusted *β* (95% CI)	*P*-value
Level 1: Maternal factors at enrolments						
Age (years)			0.06 (−0.03, 0.15)	0.178	0.02 (−0.07, 0.12)	0.604
Household socioeconomic index (*n* = 1104)			0.23 (−0.16, 0.63)	0.245		
Parity			0.04 (−0.23, 0.31)	0.751		
Body mass index (kg/m^2^) (*n* = 1110)			0.27 (0.13, 0.42)	<0.001	**0.26 (0.11, 0.40)**	**<0.001**
Education status						
None (*n* = 28)		104.5 (8.7)	−0.54 (−3.65, 2.56)		**−0.62 (−3.77, 2.53)**	
Primary (*n* = 542)		105.0 (7.7)	Reference		**Reference**	
Senior (*n* = 438)		106.5 (8.2)	1.45 (0.42, 2.48)		**1.43 (0.39, 2.47)**	
Tertiary (*n* = 109)		108.2 (9.8)	3.19 (1.51, 4.87)	<0.001	**3.14 (1.45, 4.84)**	**<0.001**
Marital status						
Single (*n* = 116)		104.7 (7.6)	−1.34 (−2.92, 0.25)			
Married/cohabiting (*n* = 967)		106.0 (8.3)	Reference			
Separated/widowed (*n* = 35)		105.3 (6.1)	−0.78 (−3.56, 1.99)	0.229		
Area of residence						
Urban (*n* = 770)		106.0 (8.3)	Reference			
Rural (*n* = 336)		105.5 (8.0)	−0.47 (−1.52, 0.59)	0.386		
Alcohol use						
No (*n* = 775)		105.8 (8.4)	Reference			
Yes (*n* = 343)		106.0 (7.8)	0.15 (−0.90, 1.19)	0.781		
Infections						
HIV						
Uninfected (*n* = 1002)		106.0 (8.3)	Reference		Reference	
Infected (*n* = 117)		104.8 (7.2)	−1.17 (−2.74, 0.41)	0.146	−0.88 (−2.48, 0.72)	0.279
Asymptomatic malaria						
Uninfected (*n* = 991)		105.8 (8.2)	Reference			
Infected (*n* = 109)		106.2 (8.6)	0.42 (−1.20, 2.05)	0.609		
* Schistosoma mansoni*						
Uninfected (*n* = 908)		105.8 (8.3)	Reference			
Infected (*n* = 204)		106.2 (7.9)	0.35 (−0.90, 1.61)	0.578		
Hookworm (*Necator americanus*)						
Uninfected (*n* = 662)		105.8 (8.1)	Reference			
Infected (*n* = 450)		105.9 (8.4)	0.10 (−0.89, 1.09)	0.844		
*Ascaris* *lumbricoides*						
Uninfected (*n* = 1084)		105.9 (8.3)	Reference			
Infected (*n* = 28)		105.7 (6.7)	−0.17 (−3.27, 2.92)	0.912		
Intervention one						
Placebo (*n* = 566)		105.5 (8.2)	Reference		Reference	
Albendazole (*n* = 553)		106.2 (8.3)	0.67 (−0.29, 1.63)	0.173	0.84 (−0.12, 1.80)	0.087
Intervention two						
Placebo (*n* = 564)		106.0 (8.1)	Reference			
Praziquantel (*n* = 555)		105.8 (8.4)	−0.20 (−1.16, 0.77)	0.686		
Level 2: Factors in childhood						
Birth weight (kg) (*n* = 932)			0.73 (−0.33, 1.80)	0.178	0.18 (−0.93, 1.29)	0.751
Sex						
Male (*n* = 583)		105.9 (7.5)	Reference		Reference	
Female (*n* = 536)		105.9 (9.0)	−0.02 (−0.98, 0.95)	0.971	0.12 (−1.18, 0.94)	0.819
Sickle-cell trait						
HbAA (*n* = 661)		106.0 (8.4)	Reference			
HbAS (*n* = 141)		105.8 (7.9)	−0.28 (−1.79, 1.23)	0.717		
Season of birth						
Dry (*n* = 651)		106.1 (8.1)	Reference			
Wet (*n* = 468)		105.5 (8.3)	−0.56 (−1.54, 0.42)	0.261		
Place of delivery						
Entebbe Hospital (*n* = 824)		105.8 (8.2)	Reference		Reference	
Home (*n* = 120)		104.9 (8.6)	−0.86 (−2.43, 0.71)		−0.37 (−3.71, 2.96)	
Others (*n* = 174)		106.8 (8.0)	0.95 (−0.39, 2.29)	0.166	0.90 (−0.87, 2.68)	0.582
Feeding status (at 6 weeks of age)						
Exclusively breast fed (*n* = 748)		106.1 (8.2)	Reference			
Mixed fed (*n* = 344)		105.4 (8.4)	−0.70 (−1.75, 0.35)			
Weaned (*n* = 14)		105.8 (7.1)	−0.28 (−4.63, 4.08)	0.430		
Intervention three						
Placebo (*n* = 553)		105.5 (8.4)	Reference			
Albendazole (*n* = 554)		106.1 (8.0)	0.61 (−0.36, 1.58)	0.218		
HIV status						
Unexposed (*n* = 1001)		106.0 (8.3)	Reference		Reference	
Exposed not infected (*n* = 100)		105.2 (7.3)	−0.83 (−2.52, 0.86)		−0.29 (−2.15, 1.57)	
Infected (*n* = 18)		102.7 (6.1)	−3.34 (−7.17, 0.49)	0.156	−3.85 (−7.81, 0.12)	0.157
Malaria infection below 5 years of age						
Clinical or asymptomatic^a^						
None (*n* = 456)		106.6 (8.0)	Reference		**Reference**	
Yes (*n* = 663)		105.3 (8.3)	−1.31 (−2.29, −0.33)	0.009	**−1.24 (−2.32, −0.17)**	**0.023**
Clinical malaria^a^						
None (*n* = 474)		106.6 (8.0)	Reference		**Reference**	
Yes (*n* = 645)		105.4 (8.3)	−1.19 (−2.17, −0.22)	0.016	**−1.08 (−2.15, −0.02)**	**0.045**
Episodes of clinical malaria^a^						
None (*n* = 474)		106.6 (8.0)	Reference		Reference	
1–2 (*n* = 382)		105.4 (8.4)	−1.13 (−2.24, −0.03)		−1.11 (−2.32, 0.11)	
≥3 (*n* = 263)		105.3 (8.2)	−1.28 (−2.52, −0.04)	0.026 [trend]	−1.05 (−2.41, 0.31)	0.133
Asymptomatic malaria^a^						
None (*n* = 983)		106.1 (8.2)	Reference		**Reference**	
Yes (*n* = 124)		103.7 (8.0)	−2.41 (−3.94, −0.88)	0.002	**−1.95 (−3.70, −0.20)**	**0.028**
*Schistosoma mansoni*						
Uninfected (*n* = 1076)		105.9 (8.2)	Reference			
Infected (*n* = 33)		104.8 (7.9)	−1.09 (−3.94, 1.76)	0.452		
*Ascaris* *lumbricoides*						
Uninfected (*n* = 1052)		105.9 (8.3)	Reference			
Infected (*n* = 57)		105.3 (7.3)	−0.62 (−2.82, 1.57)	0.576		
Hookworm (*Necator americanus*)						
Uninfected (*n* = 1085)		105.9 (8.2)	Reference			
Infected (*n* = 24)		103.8 (8.9)	−2.06 (−5.38, 1.27)	0.225		
*Trichuris* *trichiura*						
Uninfected (*n* = 997)		105.9 (8.2)	Reference			
Infected (*n* = 112)		105.6 (8.6)	−0.28 (−1.89, 1.33)	0.731		
Microfilaria (*Mansonella perstans*)						
Uninfected (*n* = 1102)		105.8 (8.2)	Reference			
Infected (*n* = 8)		109.4 (8.9)	3.58 (−2.13, 9.28)	0.219		
Level 3: Factors in adolescence						
Age (years)			2.12 (1.17, 3.08)	<0.001	**1.35 (0.32, 2.39)**	**0.009**
Body mass index (kg/m^2^)			1.27 (1.02, 1.51)	<0.001	**0.78 (0.42, 1.14)**	**<0.001**
Waist circumference (cm)			0.46 (0.36, 0.55)	<0.001	**0.21 (0.08, 0.35)**	**0.002**
Family history						
High blood pressure						
No (*n* = 1000)		105.7 (8.1)	Reference		**Reference**	
Yes (*n* = 105)		107.6 (8.3)	1.88 (0.24, 3.52)	0.025	**1.84 (0.12, 3.56)**	**0.034**
Diabetes						
No (*n* = 927)		105.8 (8.0)	Reference			
Yes (*n* = 186)		106.4 (9.2)	0.69 (−0.61, 1.99)	0.296		
Body composition analysis^c^						
Fat mass index^b^ (kg/m^2^) (*n* = 176)			3.27 (2.29, 4.24)	<0.001	1.50 (−0.38, 3.38)	0.089
Fat-free mass index^b^ (kg/m^2^) (*n* = 176)			1.54 (0.65, 2.43)	0.001	−0.86 (−2.25, 0.54)	0.188
Total body water index^b^ (kg/m^2^) (*n* = 176)			4.20 (2.97, 5.42)	<0.001	2.51 (−0.24, 5.27)	0.052
Adding salt to cooked food at the table						
No (*n* = 20)		106.2 (7.3)	0.36 (−3.28, 4.00)			
Yes (*n* = 1086)		105.9 (8.2)	Reference	0.846		
Days a fruit is eaten/week						
0–2 (*n* = 543)		106.3 (8.0)	Reference		Reference	
3–7 (*n* = 541)		105.5 (8.5)	−0.83 (−1.82, 0.15)	0.098	−0.83 (−1.84, 0.19)	0.106
Days vegetables eaten/week						
0–2 (*n* = 461)		106.4 (8.2)	Reference		**Reference**	
3–7 (*n* = 635)		105.5 (8.3)	−0.94 (−1.93, 0.05)	0.063	**−1.13 (−2.15, −0.10)**	**0.029**
Days animal-protein eaten/week						
0–2 (*n* = 726)		105.4 (7.8)	Reference		Reference	
3–7 (*n* = 374)		106.6 (8.8)	1.17 (0.16, 2.19)	0.024	0.99 (−0.06, 2.04)	0.062
Days sugared drinks taken/week						
None (*n* = 427)		105.4 (8.1)	Reference		Reference	
1–3 (*n* = 492)		105.9 (8.0)	0.54 (−0.53, 1.61)		−0.05 (−1.14, 1.05)	
4–7 (*n* = 174)		107.2 (9.1)	1.81 (0.36, 3.26)	0.051	0.96 (−0.53, 2.44)	0.358
Days a fruit is eaten/week			−0.05 (−0.27, 0.18)	0.687		
Days vegetables eaten/week			−0.18 (−0.39, 0.03)	0.085	−0.19 (−0.40, 0.03)	0.081
Days animal-protein eaten/week			0.21 (−0.07, 0.50)	0.138	0.10 (−0.20, 0.39)	0.502
Days starchy foods eaten/week			0.14 (−0.45, 0.73)	0.636		
Days sugared drinks taken/week			0.23 (0.00, 0.46)	0.049	0.11 (−0.12, 0.35)	0.325
Breast development (girls only)^b^						
Pre-pubertal (*n* = 178)		103.9 (7.8)	Reference		Reference	
Pubertal (*n* = 97)		108.0 (10.5)	4.07 (1.87, 6.26)	<0.001	1.17 (−1.26, 3.59)	0.318
Pubic hair development^b^						
Pre-pubertal (*n* = 441)		104.7 (7.4)	Reference		Reference	
Pubertal (*n* = 170)		106.5 (9.3)	1.83 (0.42, 3.24)	0.011	0.51 (−0.96, 1.98)	0.486
Snoring						
No (*n* = 932)		105.8 (8.2)	Reference			
Yes (*n* = 163)		106.3 (8.2)	0.53 (−0.83, 1.90)	0.444		
Duration of night sleep						
<9 hours (*n* = 306)		106.1 (8.0)	Reference			
9 hours (*n* = 382)		105.8 (8.8)	−0.28 (−1.51, 0.96)			
>9 hours (*n* = 405)		105.7 (7.7)	−0.39 (−1.61, 0.83)	0.818		
Smoking in household						
No (*n* = 962)		106.0 (8.3)	Reference		Reference	
Yes (*n* = 147)		104.9 (7.5)	−1.03 (−2.46, 0.40)	0.157	−0.65 (−2.10, 0.80)	0.372
Type of school attended						
Day (*n* = 117)		105.7 (7.9)	Reference		Reference	
Boarding school (*n* = 719)		107.5 (10.3)	1.76 (0.19, 3.34)	0.038	0.28 (−1.38, 1.95)	0.733
Involved in physical education at school						
No (*n* = 385)		105.5 (8.5)	Reference			
Yes (*n* = 719)		106.0 (8.1)	0.48 (−0.54, 1.50)	0.360		
Infections at the time of blood pressure measurement						
Asymptomatic malaria						
Uninfected (*n* = 1067)		106.0 (8.2)	Reference		Reference	
Infected (*n* = 22)		103.1 (9.3)	−2.85 (−6.31, 0.61)	0.106	−1.50 (−5.02, 2.02)	0.397
* Schistosoma mansoni*						
Uninfected (*n* = 964)		105.9 (8.3)	Reference			
Infected (*n* = 112)		105.7 (8.4)	−0.25 (1.88, 1.38)	0.764		
Hookworm (*Necator americanus*)						
Uninfected (*n* = 1066)		105.9 (8.3)	Reference			
Infected (*n* = 10)		103.8 (10.0)	−2.10 (−7.27, 3.07)	0.425		
Ascaris (*Ascaris lumbricoides*)						
Uninfected (*n* = 1073)		105.9 (8.3)	Reference		Reference	
Infected (*n* = 3)		98.7 (1.6)	−7.34 (−16.65, 2.17)	0.132	−7.04 (−15.97, 1.88)	0.117
*Trichuris trichiura*						
Uninfected (*n* = 1036)		105.8 (8.3)	Reference		**Reference**	
Infected (*n* = 40)		107.9 (8.3)	2.16 (−0.46, 4.78)	0.106	**3.48 (0.79, 6.18)**	**0.010**

Model building followed the hierarchical approach, adding factors sequentially at three levels starting with the distal factors (level 1). Factors at the same level were added to the model at the same time and considered confounders for each other and for proximal factors. A *P*-value < 0.20 was used for considering the inclusions and maintenance of factors in the model

Adjusted *β* with 95% CI excluding 0 in bold

*β* linear regression coefficient: mean difference in blood pressure (BP) measured in mmHg

^a^Not included in the model together but each was adjusted for all other model variables

^b^Not included in multivariable model building for other exposures because of large proportion of missing information but each was adjusted for variables in the final model building

^c^Not adjusted for body mass index because body mass index is on the causal pathway

**Table 2:** Factors investigated for association with diastolic blood pressure among adolescents from the Entebbe Mother and Baby Study (*N* = 1119)

**Factors**	Mean BP (SD)	Crude *β* (95% CI)	*P*-value	Adjusted *β* (95% CI)	*P*-value
Level 1: Maternal factors					
Age (years)		0.08 (−0.00, 0.15)	0.058	0.05 (−0.03, 0.13)	0.247
Household socioeconomic index (*n* = 1104)		0.22 (−0.13, 0.56)	0.225		
Parity		0.08 (−0.16, 0.32)	0.530		
Body mass index (*n* = 1110)		0.16 (0.03, 0.29)	0.014	**0.14 (0.01, 0.27)**	**0.030**
Education status					
None (*n* = 28)	65.1 (9.3)	0.44 (−2.32, 3.19)		**0.08 (−2.71, 2.89)**	
Primary (*n* = 542)	64.6 (6.7)	Reference		**Reference**	
Senior (*n* = 438)	65.5 (7.5)	0.92 (0.01, 1.84)		**1.00 (0.07, 1.92)**	
Tertiary (*n* = 109)	66.8 (8.0)	2.14 (0.65, 3.64)	0.023	**2.08 (0.57, 3.59)**	**0.022**
Marital status					
Single (*n* = 116)	64.2 (6.4)	−1.19 (−2.59, 0.21)		−1.26 (−2.69, 0.16)	
Married/cohabiting (*n* = 967)	65.4 (7.4)	Reference		Reference	
Separated/widowed (*n* = 35)	63.5 (6.0)	−1.91 (−4.36, 0.54)	0.089	−1.91 (−4.38, 0.54)	0.075
Area of residence					
Urban (*n* = 770)	65.3 (7.5)	Reference			
Rural (*n* = 336)	64.9 (6.8)	0.49 (−1.42, 0.44)	0.302		
Alcohol use					
No (*n* = 775)	65.3 (7.5)	Reference			
Yes (*n* = 343)	65.0 (6.6)	−0.34 (−1.26, 0.59)	0.477		
Infections					
HIV					
Uninfected (*n* = 1002)	65.2 (7.3)	Reference			
Infected (*n* = 117)	64.9 (6.5)	−0.35 (−1.74, 1.05)	0.626		
Asymptomatic malaria					
Uninfected (*n* = 991)	65.2 (7.4)	Reference			
Infected (*n* = 109)	64.9 (6.6)	−0.29 (−1.73, 1.15)	0.695		
*Schistosoma mansoni*					
Uninfected (*n* = 908)	65.2 (7.1)	Reference			
Infected (*n* = 204)	65.5 (7.7)	0.31 (−0.79, 1.41)	0.579		
Hookworm (*Necator americanus*)					
Uninfected (*n* = 662)	65.1 (7.1)	Reference			
Infected (*n* = 450)	65.4 (7.4)	0.27 (−0.60, 1.14)	0.539		
*Ascaris lumbricoides*					
Uninfected (*n* = 1084)	65.3 (7.3)	Reference			
Infected (*n* = 28)	65.1 (5.5)	−0.18 (−2.90, 2.54)	0.896		
Intervention one					
Placebo (*n* = 566)	65.0 (6.9)	Reference			
Albendazole (*n* = 553)	65.4 (7.7)	0.39 (−0.46, 1.24)	0.366		
Intervention two					
Placebo (*n* = 564)	65.4 (7.3)	Reference			
Praziquantel (*n* = 555)	65.0 (7.2)	−0.44 (−1.29, 0.42)	0.315		
Level 2: Factors in childhood					
Birth weight (kg) (*n* = 932)		0.66 (−0.27, 1.59)	0.164	0.57 (−0.40, 1.53)	0.246
Sex					
Male (*n* = 583)	64.9 (7.2)	Reference		Reference	
Female (*n* = 536)	65.5 (7.4)	0.64 (−0.21, 1.49)	0.141	0.49 (−0.43, 1.42)	0.294
Sickle-cell trait					
HbAA (*n* = 661)	65.4 (7.1)	Reference			
HbAS (*n* = 141)	65.5 (7.4)	0.15 (−1.16, 1.46)	0.825		
Season of birth					
Dry (*n* = 651)	65.5 (7.3)	Reference		Reference	
Wet (*n* = 468)	64.7 (7.2)	−0.79 (−1.65, 0.07)	0.073	0.59 (−1.52, 0.35)	0.214
Place of delivery					
Entebbe Hospital (*n* = 824)	65.1 (7.1)	Reference			
Home (*n* = 120)	65.4 (8.5)	0.36 (−1.03, 1.76)			
Others (*n* = 174)	65.7 (7.3)	0.61 (−0.58, 1.80)	0.564		
Feeding status (at 6 week of age)					
Exclusive breast fed (*n* = 748)	65.4 (7.4)	Reference			
Mixed fed (*n* = 344)	64.7 (7.0)	−0.63 (−1.56, 0.30)			
Weaned (*n* = 14)	67.1 (4.4)	1.78 (−2.07, 5.63)	0.251		
Intervention three					
Placebo (*n* = 553)	64.9 (7.0)	Reference		Reference	
Albendazole (*n* = 554)	65.5 (7.5)	0.62 (−0.24, 1.47)	0.156	0.56 (−0.37, 1.48)	0.233
HIV status					
Unexposed (*n* = 1001)	65.2 (7.3)	Reference			
Exposed not infected (*n* = 100)	65.1 (6.7)	−0.12 (−1.62, 1.37)			
Infected (*n* = 18)	63.5 (5.1)	−1.71 (−5.10, 1.68)	0.609		
Malaria infection below 5 years of age					
Clinical or asymptomatic malaria^a^					
No (*n* = 456)	65.9(7.1)	Reference		**Reference**	
Yes (*n* = 663)	64.6 (7.3)	−1.28 (−2.14, −0.41)	0.004	**−1.47 (−2.41, −0.53)**	**0.002**
Clinical malaria^a^					
None (*n* = 474)	66.0 (7.2)	Reference		**Reference**	
Yes (*n* = 645)	64.6 (7.3)	−1.38 (−2.24, −0.51)	0.002	**−1.33 (−2.26, −0.39)**	**0.005**
Episodes of clinical malaria^a^					
None (*n* = 474)	65.9 (7.2)	Reference		**Reference**	
1–2 (*n* = 382)	64.5 (7.3)	−1.45 (−2.42, −0.47)		**−1.53 (−2.59, −0.46)**	
≥3 (*n* = 263)	64.9 (7.4)	−1.02 (−2.12, 0.07)	0.011	**−1.03 (−2.22, 0.16)**	**0.015**
Asymptomatic malaria^a^					
None (*n* = 983)	64.5 (7.3)	Reference		Reference	
Yes (*n* = 124)	64.9 (7.4)	−1.45 (−2.80, −0.10)	0.035	−1.35 (−2.89, 0.18)	0.082
*Schistosoma mansoni*					
Uninfected (*n* = 1076)	65.2 (7.3)	Reference			
Infected (*n* = 33)	64.5 (5.8)	0.67 (−3.18, 1.84)	0.602		
*Ascaris* *lumbricoides*					
Uninfected (*n* = 1052)	65.2 (7.3)	Reference			
Infected (*n* = 57)	64.5 (7.1)	−0.75 (−2.68, 1.18)	0.445		
Hookworm (*Necator americanus*)					
Uninfected (*n* = 1085)	65.2 (7.3)	Reference		Reference	
Infected (*n* = 24)	62.9 (5.8)	−2.29 (−5.22, 0.64)	0.125	−1.79 (−4.93, 1.35)	0.261
*Trichuris* *trichiura*					
Uninfected (*n* = 997)	65.1 (7.2)	Reference			
Infected (*n* = 112)	65.8 (7.7)	0.67 (−0.74, 2.09)	0.353		
Microfilaria (*Mansonella perstans*)					
Uninfected (*n* = 1102)	65.1 (7.2)	Reference			
Infected (*n* = 8)	67.3 (3.3)	2.12 (−2.91, 7.14)	0.409		
Level 3: Factors in adolescence					
Age (years)		1.85(1.00, 2.70)	<0.001	**1.53 (0.63, 2.43)**	**<0.001**
Body mass index (kg/m^2^)		0.28 (0.20, 0.36)	<0.001	**0.74 (0.42, 1.05)**	**<0.001**
Waist circumference (cm)		0.88 (0.66, 1.10)	<0.001	0.07 (−0.05, 0.18)	0.279
Family history					
High blood pressure					
No (*n* = 1000)	65.0 (7.2)	Reference		**Reference**	
Yes (*n* = 105)	66.7 (7.6)	1.65 (0.19, 3.12)	0.027	**1.57 (0.08, 3.06)**	**0.037**
Diabetes					
No (*n* = 927)	65.2 (7.2)	Reference			
Yes (*n* = 186)	65.5 (7.8)	0.35 (−0.80, 1.49)	0.553		
Body composition analysis^c^					
Fat mass index^b^ (kg/m^2^) (*n* = 176)		1.75 (0.83, 2.69)	<0.001	0.87 (−0.73, 2.47)	0.255
Fat-free mass index^b^ (kg/m^2^) (*n* = 176)		1.19 (0.40, 1.98)	0.003	0.28 (−0.90, 1.45)	0.622
Total body water index^b^ (kg/m^2^) (*n* = 176)		2.13 (0.95, 3.30)	<0.001	1.51 (−0.86, 3.88)	0.180
Adding salt to cooked food at the table					
No (*n* = 20)	67.4 (6.1)	2.19 (−1.04, 5.41)		2.72 (−0.39, 5.82)	
Yes (*n* = 1086)	65.2 (7.3)	Reference	0.184	Reference	0.083
Days a fruit is eaten/week					
0–2 (*n* = 543)	65.7 (7.1)	Reference		**Reference**	
3–7 (*n* = 541)	64.7 (7.5)	−0.98 (−1.85, −0.11)	0.028	**−0.96 (−1.83, −0.10)**	**0.027**
Days vegetables eaten/week					
0–2 (*n* = 461)	65.4 (7.1)	Reference			
3–7 (*n* = 635)	65.1 (7.5)	−0.27 (−1.15, 0.60)	0.540		
Days animal-protein eaten/week					
0–2 (*n* = 726)	65.1 (6.9)	Reference			
3–7 (*n* = 374)	65.4 (8.0)	0.30 (−0.61, 1.20)	0.523		
Days sugared drinks taken/week					
None (*n* = 427)	65.0 (7.1)	Reference		Reference	
1–3 (*n* = 492)	65.2 (7.4)	0.25 (−0.70, 1.20)		0.12 (−0.84, 1.08)	
4–7 (*n* = 174)	66.0 (7.5)	1.06 (−0.23, 2.35)	0.271	0.54 (−0.75, 1.83)	0.707
Days a fruit is eaten/week					
Days vegetables eaten/week		0.02 (−0.16, 0.1)	0.800		
Days animal-protein eaten/week		0.14 (−0.11, 0.39)	0.284		
Days starchy foods eaten/week		0.03 (−0.50, 0.55)	0.924		
Days sugared drinks taken/week		0.20 (0.00, 0.41)	0.048		
Breast development (girls only)^b^					
Pre-pubertal (*n* = 178)	64.1 (6.1)	Reference		Reference	
Pubertal (*n* = 97)	67.2 (7.9)	3.067 (1.38, 4.76)	<0.001	0.98 (−0.88, 2.84)	0.281
Pubic hair development^b^					
Pre-pubertal (*n* = 441)	64.1 (6.6)	Reference		Reference	
Pubertal (*n* = 170)	66.1 (7.6)	2.04 (0.82, 3.26)	0.001	0.68 (−0.62, 1.99)	0.293
Snoring					
No (*n* = 932)	65.1 (7.2)	Reference			
Yes (*n* = 163)	65.6 (7.8)	0.44 (−0.78, 1.66)	0.477		
Duration of night sleep					
<9 hours (*n* = 306)	65.8 (7.6)	Reference		Reference	
9 hours (*n* = 382)	64.8 (7.1)	−1.03 (−2.11, 0.06)		−0.92 (−2.02, 0.18)	
>9 hours (*n* = 405)	65.2 (7.2)	−0.79(−1.86, 0.28)	0.160	−0.67 (−1.76, 0.43)	0.240
Smoking in household					
Non (*n* = 962)	65.2 (7.3)	Reference			
Yes (*n* = 147)	65.0 (6.8)	−0.21 (−1.46, 1.06)	0.745		
Type of school attended					
Day (*n* = 117)	65.1 (7.2)	Reference		Reference	
Boarding school (*n* = 719)	66.2 (7.8)	1.13 (−0.26, 2.52)	0.112	−0.24 (−1.67, 1.20)	0.737
Involved in physical education at school					
No (*n* = 385)	65.0 (6.9)	Reference			
Yes (*n* = 719)	65.3 (7.5)	0.32 (−0.58, 1.22)	0.482		
Infections at the time of blood pressure measurement					
Asymptomatic malaria					
Uninfected (*n* = 1067)	65.3 (7.3)	Reference			
Infected (*n* = 22)	64.0 (5.5)	−1.31 (−4.36, 1.75)	0.401		
*Schistosoma mansoni*					
Uninfected (*n* = 964)	65.2 (7.4)	Reference			
Infected (*n* = 112)	65.0 (5.8)	−0.19 (−1.62, 1.24)	0.791		
Hookworm (*Necator americanu*s)					
Uninfected (*n* = 1066)	65.2 (7.3)	Reference			
Infected (*n* = 10)	64.0 (5.9)	−1.25 (−5.80, 3.30)	0.590		
Ascaris (*Ascaris lumbricoides*)					
Uninfected (*n* = 1073)	65.2 (7.3)	Reference			
Infected (*n* = 3)	62.3 (4.3)	−2.86 (−11.14, 5.42)	0.498		
*Trichuris trichiura*					
Uninfected (*n* = 1036)	65.1 (7.2)	Reference			
Infected (*n* = 40)	66.4 (9.4)	1.23 (−1.07, 3.54)	0.294		

Model building followed the hierarchical approach, adding factors sequentially at three levels starting with the distal factors (level 1). Factors at the same level were added to the model at the same time and considered confounders for each other and for proximal factors. A *P*-value < 0.20 was used for considering the inclusion and maintenance of factors in the model

Adjusted *β* for which 95% CI exclude 0 are highlighted in bold

*β* linear regression coefficient: mean difference in blood pressure (BP) measured in mmHg

^a^Not included in the model together but each was adjusted for all other variables in the model

^b^Not included in multivariable model building for other exposures because of large proportion of missing information; but each was adjusted for variables in the final model building

^c^Not adjusted for body mass index because body mass index is on the causal pathway

Characteristics at the time of BP measurement showing a crude positive association with systolic BP were age, BMI, waist circumference, family history of high BP, body composition variables and puberty stage covariates. In multivariable analysis, systolic BP increased, on average, by 1.35 mmHg, 95% CI (0.32, 2.39) for each 1-year increase in adolescents’ age; by 0.78 mmHg (0.42, 1.14) per unit increase in BMI; and by 0.21 mmHg (0.08, 0.35) per centimetre increase in waist circumference. Family history of high BP remained associated with increased systolic BP, *β* = 1.84 (0.12, 3.56) after adjustment for maternal and childhood factors. Body composition and puberty stage covariates were no longer associated with systolic BP on adjusting for adolescents’ age, BMI and waist circumference.

Lifestyle factors crudely associated with increased systolic BP were increased animal-protein consumption, increased consumption of sugared drinks and attending a boarding school rather than a day school. Increased fruit and vegetable consumption were associated with reduced systolic BP. After adjusting for confounders, systolic BP reduced on average by 1.13 mmHg (−2.15, −0.10) among adolescents who consumed vegetables for 3–7 days/week (versus 0–2 days/week).

Current infection with *Trichuris trichiura* was positively associated with systolic BP after adjusting for confounders (*β* = 3.48 mmHg (0.79, 6.18)). Systolic BP dropped by 1.24 mmHg (−2.32, −0.17) among adolescents who had malaria in childhood compared to those who had not. Both clinical and asymptomatic malaria were independently associated with lower BP in multivariable analysis. Weight and height at 10 and 11 years of age were reduced among adolescents with childhood clinical and or asymptomatic malaria (Supplementary Table 1). Compared to those with no asymptomatic malaria, having asymptomatic malaria in childhood was associated with, on average, a 3.2 cm reduction in height, 95% CI (−4.5, −2.0) and a 2.1 kg reduction in weight, 95% CI (−3.0, −1.9). The effect of childhood malaria on adolescent BP was weaker on adjusting for adolescent BMI (Supplementary Table 2).

Genetic data were available for 802 (72%) participants of whom 141 (18%) had sickle-cell trait (HbAS) and 661 (82%) normal haemoglobin (HbAA). Sickle-cell trait was not associated with systolic BP (*β* = −0.28 mmHg (−1.79, 1.23)), even after adjusting for age and sex. HbAS was inversely associated with malaria (Supplementary Table 3): in those with HbAA, 63% had clinical or asymptomatic malaria up to 5 years compared to 51% with HbAS (*P-* value = 0.008).

Findings for diastolic BP were broadly similar to those for systolic BP, with the exceptions that higher fruit rather than vegetable consumption was associated with lower diastolic BP, and there was no association with waist circumference or Trichuris infection. No associations were observed between adolescent BP and any of the other factors considered in this population (Tables 1 and 2).

## Discussion

Persistent high BP and pre-hypertension were unusual in early adolescence in this setting. Maternal gestational BMI and education status at enrolment, participant’s family history of hypertension, and adolescents’ age and BMI at BP measurement were positively associated with both systolic BP and diastolic BP. Malaria parasitaemia in childhood, and increased vegetable and fruit consumption were inversely associated with systolic BP and diastolic BP, respectively. Concurrent Trichuris infection was positively associated with systolic BP but not with diastolic BP. There were no effects of anti-helminth trial interventions (in pregnancy or childhood) on adolescent BP and no associations between prior helminth infection (in pregnancy or childhood) and adolescent BP.

Our findings are consistent with several earlier studies [25, 26]. We have shown that consuming vegetables and fruits for 3–7 days/week was associated with lower systolic BP and diastolic BP, respectively. Our results support findings from a cross-sectional study that consuming fruits and vegetables (>400 g/day) lowers systolic BP and diastolic BP in adults [26]. We have shown a positive association of BP with maternal gestational BMI, and adolescent BMI and waist circumference at the time of BP measurement, consistent with earlier studies [13].

Malaria parasitaemia in childhood was associated with lower BP in early adolescence, consistent with findings from a cross-sectional study among 5–18-year-old Ugandan students, which reported that current asymptomatic malaria was associated with lower BP [25]. Our study was underpowered to detect the effect of current parasitaemia on BP, with only 22 (2.1%) adolescents had parasitaemic at the time of BP measurement.

Sub-microscopic malaria was most likely misclassified as negative in this population, since in malaria-endemic areas, asymptomatic malaria often presents as sub-microscopic in individuals with past malaria infection [27]. We found no association between sickle-cell trait and adolescent BP; contrary to the hypothesis advanced by Etyang et al., who used sickle-cell trait as an instrumental variable in a Mendelian randomisation study [28]. In the predominantly adult populations from Kenya, sickle-cell trait (linked with partial protection against malaria) was associated with lower BP in Kilifi (currently a low-moderate but historically a high malaria transmission area) compared to Nairobi (no malaria transmission) [29]. The differences in malaria exposure intensity and participant age distribution between our study and the Kenyan study could explain our contrasting results.

Similar to earlier studies [30, 31], childhood malaria was associated with reductions in both weight and height, and some of the inverse association seen in this study may be explained by this mechanism, or by confounding by unmeasured factors. The escalating burden of high BP has coincided with the declining malaria burden on the African continent [2, 32, 33]. This could be explained by the epidemiological transition process on continent, or the effect could be more direct; the mechanisms remain to be elucidated.

Current but not previous infection with *Trichuris trichiura* (a type of soil transmitted helminth, commonly known as whipworm) was associated with increased systolic BP in early adolescence. To our knowledge, no study has previously reported such an association. This may reflect short-term effects (probably arterial stiffness from inflammatory reaction) or it could be a spurious finding due to the many exposures included in the analysis. The effect of current *Trichuris trichiura* infection on BP is likely not mediated through increasing BMI (weight or height); there was no difference in these measures between adolescents with and without current *Trichuris trichiura* infection.

Unlike previous studies [34], we found no association between BP and salt intake. The lack of evidence for this relationship in our study could be due to measurement error from self-report, or the fact that nearly everyone added salt to cooked food. Measuring sodium in a 24-h urine sample or in commonly consumed local foods would provide a more accurate reflection of daily intake. Physical activity was not associated with lower BP, contrary to earlier literature [35]; sedentary lifestyles are still fairly uncommon in this population.

Previous studies have linked hypertension to socioeconomic determinants (socioeconomic status (SES), education, income, urbanisation) [12, 36]. Our study is consistent with a Uganda study in adults which showed that BP was not associated with urban residence [37] but contrary to studies linking increased BP with low SES [36] and urbanisation [12]. We have shown that higher maternal education was associated with increased BP in adolescents, whereas other studies, predominantly from high-income countries, report an inverse association [36]. Although low SES and education is associated with hypertension in the developed world [36], the relationship may be inverse in less developed countries [38]. In these settings, offspring from more highly educated households are more likely to have sedentary lifestyles and unhealthy dietary practices, and to be obese, compared to offspring from less-educated households.

Strengths of this study included its longitudinal design with prospectively collected data reducing recall and reporter bias, the use of robust BP procedures and the measurement of BP on up to two extra occasions in those with BP ≥95th percentile at the initial visit, to avoid overestimation of high BP. It is unlikely that white-coat phenomenon was an issue as participants regularly attend this clinic for scheduled and/or illness visits. The use of digital machines reduced differences in BP reading between operators which can occur with auscultation.

Study limitations include the possibility of residual confounding by unmeasured factors (such as glomerular filtration rate (GFR)). The GFR could not be estimated as creatinine was only measured for a subgroup of the participants. The use of digital BP machines may overestimate BP; however, digital devices used in this study were calibrated twice annually. A large number of statistical tests were undertaken; thus, some findings may be due to multiplicity. However, it is reassuring that most findings are consistent with previous literature, albeit from different settings. Not inviting all adolescents (those with pre-hypertension or normal BP on day 1) for up to two extra BP measurements might have resulted in an underestimation in the overall prevalence of pre-hypertension and hypertension. We modelled BP as a continuous outcome, since analysing high or pre-hypertensive BP versus normal BP as a binary outcome (or outcomes) would be underpowered, consequently our findings may not necessarily reflect associations with hypertensive disease.

In summary, routine BP screening which is seldom conducted for adolescents at health care visits remains vital in the control and prevention of CVDs later in life. Similar life-course factors to those observed in high-income settings (such as adiposity and diet) affect both systolic BP and diastolic BP among African adolescents. Interventions during pregnancy, childhood and early adolescence could be vital in the control and prevention of later high BP. Multiple intervention strategies initiated during pregnancy and the early postnatal period and continued across a lifetime could be fundamental in the control of adulthood hypertension and CVDs.

### Summary

#### What is known about the topic

- High blood pressure and cardiovascular diseases are increasing in Africa.
- Scarcity of data on blood pressure risk factors among African children and adolescents.
- The risk factors for high blood pressure may differ from those seen in high-income non-tropical settings.

#### What this paper adds

- Malaria infection in childhood is associated with reduced blood pressure among adolescents. Effects of childhood malaria on later blood pressure may be partially mediated through chronic reduction in weight and height.
- Current infection with *Trichuris trichiura* is associated with increased blood pressure.
- Interventions during pregnancy, childhood and early adolescence could be vital in the prevention of high blood pressure later in life.

## Supplementary information

Supplementary tables
