# Supplementary material for: Blood pressure risk factors in early adolescents: results from a Ugandan birth cohort
Source: J Hum Hypertens. 2019 Feb 25;33(9):679–92. doi: 10.1038/s41371-019-0178-y (PMC6760975; doi:10.1038/s41371-019-0178-y)
Supplement: Supplementary file 1 — Supplementary tables [file 41371_2019_178_MOESM1_ESM.docx]

| **Table S1:** | | | **Relationship between infection and anthropometry at 10 and 11 years of age** | | | | | | |
| --- | --- | --- | --- | --- | --- | --- | --- | --- | --- |
| **Malaria infection in childhood**  **(≤ 5 years of age)** | | | | **Weight (kg)** | |  | | **Height (cm)** | |
|  |  |  |  | **Mean (95% CI)** | **p-value** |  | | **Mean (95% CI)** | **p-value** |
| Clinical or asymptomatic malaria | | | |  |  | |  | |  |
|  |  | Uninfected (n=456) | | 30.2 (29.7, 30.7) |  | | 136.7 (136.0, 137.4) | |  |
|  |  | Infected (663) | | 29.1 (28.7, 29.4) |  | | 135.0 (134.4, 135.5) | |  |
|  | Difference (Uninfected-infected) | | | 1.2 (0.5, 1.8) | <0.001 | | 1.7 (0.9, 2.6) | | <0.001 |
|  | Clinical malaria | | |  |  | |  | |  |
|  |  | Uninfected (n=474) | | 30.2 (29.7, 30.7) |  | | 136.7 (136.0, 137.3) | |  |
|  |  | Infected (n=645) | | 29.1 (28.7, 29.5) |  | | 134.9 (134.4, 135.4) | |  |
|  | Difference (Uninfected-infected) | | | 1.1 (0.5, 1.7) | <0.001 | | 1.7 (0.9, 2.5) | | <0.001 |
|  | Asymptomatic malaria | | |  |  | |  | |  |
|  |  | Uninfected (n=983) | | 29.8 (29.4, 30.1) |  | | 136.0 (135.6, 136.50 | |  |
|  |  | Infected (n=124) | | 27.7 (26.9, 28.5) |  | | 132.8 (131.7, 133.9) | |  |
|  | Difference (Uninfected-infected) | | | 2.1 (1.1, 3.0) | <0.001 | | 3.3 (2.0, 4.5) | | <0.001 |
| **Current Trichuris infection** | | | |  |  | |  | |  |
|  |  | Uninfected (1,036) | | 29.5 (29.2, 29.9) |  | | 135.7 (135.3, 136.1) | |  |
|  |  | Infected (n=40) | | 29.4 (28.2, 30.7) |  | | 133.5 (131.6, 135.5) | |  |
|  | Difference (Uninfected-infected) | | | 0.1 (-1.5, 1.8) | 0.900 | | 2.2 (0.0, 4.4) | | 0.049 |

| **Table S2.** | | | | **Association between malaria and blood pressure among adolescents from the Entebbe Mother and Baby Study (N=1119)** | | | | | | | |
| --- | --- | --- | --- | --- | --- | --- | --- | --- | --- | --- | --- |
| **Systolic blood pressure** | | | | | |  |  | |  |  |  |
|  | **Childhood malaria**  **(≤ 5 years of age)** | | | | **Mean blood pressure (SD)** | | | **Crude**  **β (95% CI)** | **P-value** | **Adjusted**  **β (95% CI) ^a^** | **P-value ^b^** |
|  |  | Clinical or asymptomatic | | | |  |  | |  |  |  |
|  |  |  | No (n=456) | | | 106.7 (8.0) | Reference | |  | Reference |  |
|  |  |  | Yes (n=663) | | | 105.3 (8.3) | -1.44 (-2.42, -0.46) | | 0.009 | -1.18 (-2.12, -0.24) | <0.001 |
|  |  | Clinical malaria | | | |  |  | |  |  |  |
|  |  |  | No (n=474) | | | 106.6 (8.0) | Reference | |  | Reference |  |
|  |  |  | Yes (n=645) | | | 105.4 (8.3) | -1.19 (-2.17, -0.22) | | 0.016 | -0.97 (-1.91, -0.04) | <0.001 |
|  |  | Episodes of clinical malaria | | | |  |  | |  |  |  |
|  |  |  | None (n=474) | | | 106.6 (8.0) | Reference | |  | Reference |  |
|  |  |  | 1-2 (n=382) | | | 105.4 (8.4) | -1.13 (-2.24, -0.03) | |  | -0.79 (-1.85, 0.28) |  |
|  |  |  | ≥3 (n=263) | | | 105.3 (8.2) | -1.28 (-2.52, -0.04) | | 0.026 [trend] | -1.24 (-2.43, -0.6) | <0.001 |
|  |  | Asymptomatic malaria | | | |  |  | |  |  |  |
|  |  |  | No (n=983) | | | 106.1 (8.2) | Reference | |  | Reference |  |
|  |  |  | Yes (n=124) | | | 103.7 (8.0) | -2.41 (-3.94, -0.88) | | 0.002 | -1.95 (-3.42, -0.48) | <0.001 |
| **Diastolic blood pressure** | | | | | | | | | | | |
|  | Clinical or asymptomatic malaria | | | | |  |  | |  |  |  |
|  |  |  | No (n=456) | | | 66.0 (7.2) | Reference | |  | Reference |  |
|  |  |  | Yes (n=663) | | | 64.6 (7.3) | -1.38 (-2.24, -0.51) | | 0.002 | -1.20 (-2.04, -0.35) | <0.001 |
|  |  | Clinical malaria | | | |  |  | |  |  |  |
|  |  |  | No (n=474) | | | 65.9 (7.2) | Reference | |  | Reference |  |
|  |  |  | Yes (n=645) | | | 64.7 (7.3) | -1.27 (-2.13, -0.41) | | 0.004 | -1.12 (-1.96, -0.28) | <0.001 |
|  |  | Episodes of clinical malaria | | | |  |  | |  |  |  |
|  |  |  | None (n=474) | | | 65.9 (7.2) | Reference | |  | Reference |  |
|  |  |  | 1-2 (n=382) | | | 64.5 (7.3) | -1.45 (-2.42, -0.47) | |  | -1.21 (-2.16, -0.25 |  |
|  |  |  | ≥3 (n=263) | | | 64.9 (7.4) | -1.02 (-2.12, 0.07) | | 0.011 | -1.00 (-2.06, 0.07) | <0.001 |
|  |  | Asymptomatic malaria | | | |  |  | |  |  |  |
|  |  |  | No (n=983) | | | 65.3 (7.3) | Reference | |  | Reference |  |
|  |  |  | Yes (n=124) | | | 63.9 (6.7) | -1.45 (-2.80, -0.10) | | 0.035 | -1.13(-2.44, 0.19) | <0.001 |
| ^a^ Adjusted for current body mass index  ^b^ Likelihood ratio test P-value | | | | | | | | | | | |

| **Table S3:** | | | **Relationship between sickle-cell trait and malaria** | | | | | | |  |
| --- | --- | --- | --- | --- | --- | --- | --- | --- | --- | --- |
| **Malaria infection in childhood (≤ 5 years of age)** | | | | **HbAA (n=661)** | |  | | **HbAS (n=141)** | |  |
|  |  |  |  | **Frequency** | **Percentage** |  | | **Frequency** | **Percentage** | **P-value** |
|  | Clinical or asymptomatic malaria | | | |  | |  | |  |  |
|  |  | No (n=447) | | 244 | 36.9 | | 69 | | 48.9 |  |
|  |  | Yes (n=663) | | 417 | 63.1 | | 72 | | 51.1 | 0.008 |
|  | Clinical malaria | | |  |  | |  | |  |  |
|  |  | None (n=474) | | 252 | 38.1 | | 70 | | 49.7 |  |
|  |  | Yes (n=645) | | 409 | 61.9 | | 71 | | 50.4 | 0.011 |
|  | Episodes of clinical malaria | | |  |  | |  | |  |  |
|  |  | None (n=474) | | 252 | 38.1 | | 70 | | 49.7 |  |
|  |  | 1-2 (n=382) | | 229 | 34.6 | | 49 | | 34.8 |  |
|  |  | ≥ 3 (n=263) | | 180 | 27.2 | | 22 | | 15.6 | 0.006 |
|  | Asymptomatic malaria | | |  |  | |  | |  |  |
|  |  | None (n=983) | | 58 | 88.1 | | 127 | | 90.1 |  |
|  |  | Yes (n=124) | | 79 | 12.0 | | 14 | | 9.9 | 0.496 |

HbAS: sickle-cell trait, HbAA: normal haemoglobin
